# Regulated to respond: dual localization and dynamic control of AVP1 in plant carbon partitioning

**DOI:** 10.3389/fpls.2025.1605111

**Published:** 2025-09-11

**Authors:** Roberto Gaxiola, Kendal D. Hirschi

**Affiliations:** ^1^ School of Life Sciences, Arizona State University, Tempe, AZ, United States; ^2^ Biological Sciences, University of Texas at El Paso, El Paso, TX, United States

**Keywords:** AVP1, pyrophosphate, stress, ubiquitination, H+-PPase, carbon partitioning

## Abstract

Arabidopsis Vacuolar Pyrophosphatase 1 (AVP1), a conserved type I proton-pumping pyrophosphatase (H^+^-PPase), was characterized as tonoplast localized with a role in acidifying the lumen and removing cytosolic pyrophosphate (PPi), a byproduct of biosynthesis. During the last several decades evidence has accumulated that AVP1also localizes to the plasma membrane, particularly in phloem companion cells, where it may function in reverse—synthesizing PPi from the proton motive force. This directional flexibility allows AVP1 to contribute to intracellular homeostasis and to phloem loading, supporting carbon partitioning from source to sink tissues. AVP1 activity is modulated post-translational through ubiquitin-dependent turnover, enabling plants to adjust in response to metabolic conditions. AVP1’s localization, regulation, and metabolic integration position it as a coordinator of energy balance. These features highlight AVP1 as a focal point for future approaches focused on crop resource-use efficiency and climate resilience.

## Introduction: a systems perspective on AVP1 function and regulation

**Figure 1:**
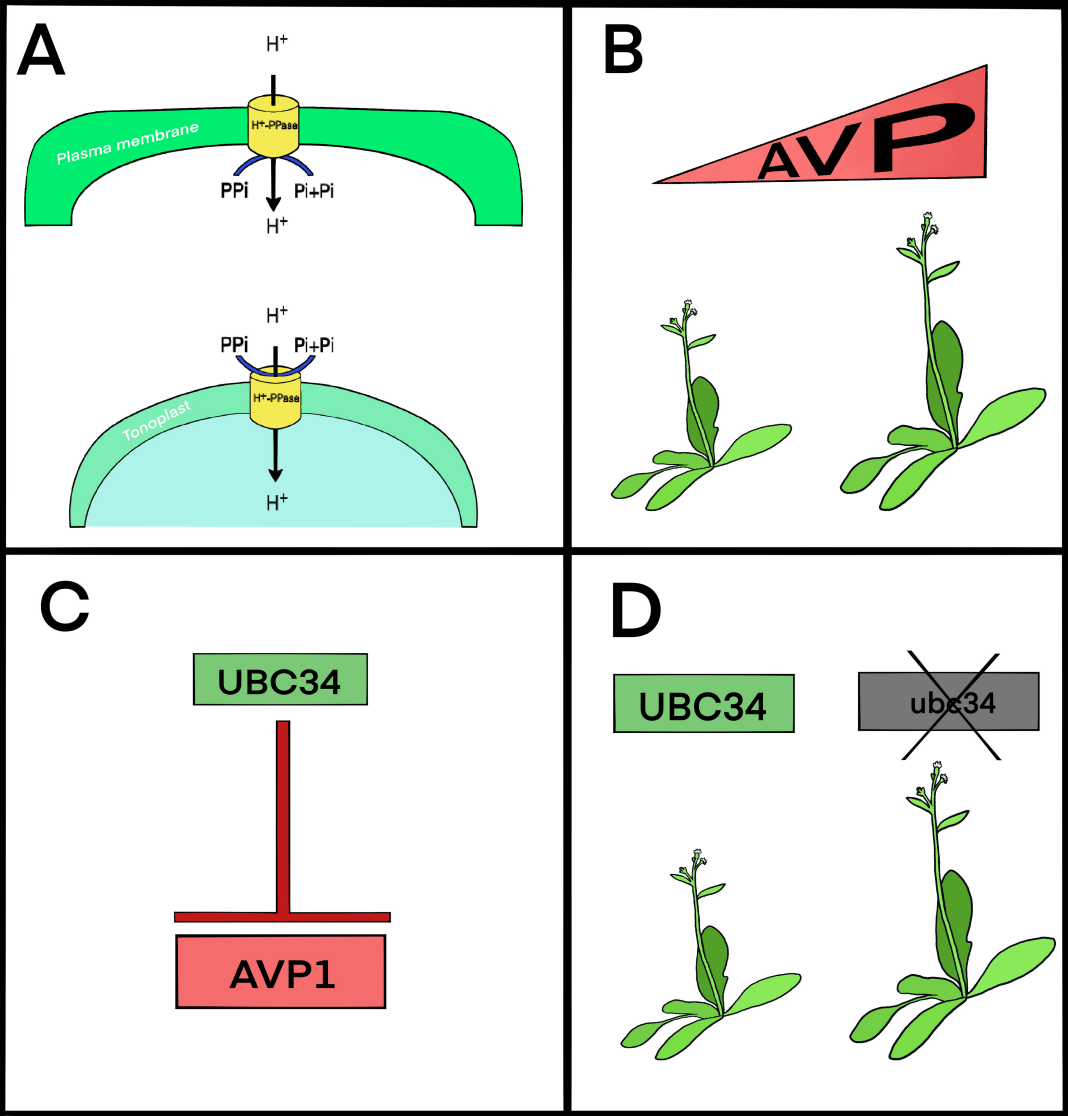
AVP1 contributes to yield through its localization, regulation, and expression. **(A)** AVP1 is found in both the vacuolar (tonoplast) and plasma membranes. Its function depends on where it’s localized within the cell. **(B)** Whether through genetic engineering or natural variation, increased expression of AVP1 (or its homologues) is associated with better plant growth and higher yields. **(C)** At the plasma membrane, AVP1 is negatively regulated by UBC34, a ubiquitin-conjugating enzyme that likely targets it for degradation. **(D)** Plants lacking UBC34 show improved yield, similar to what’s observed when AVP1 expression is elevated.

Optimizing carbon allocation and energy efficiency in plants remains a worthy goal. The H^+^-pyrophosphatase AVP1 is a promising candidate for engineering crops for increased biomass and stress adaptation. In this review, we explore how AVP1 operates at the nexus of energy metabolism, developmental plasticity, and source–sink coordination. We begin by revisiting its vacuolar function, then examine evidence for its plasma membrane localization and reverse catalytic activity (**Figure 1A**). We outline here how, for decades, increased expression of AVP1 has been shown to enhance yield (**Figure 1B**). More recently, natural variation in AVP1 expression has also been linked to differences in biomass accumulation (Schilling et al., 2017). The impetus for this review is to highlight recent insights into post-translational regulation via UBC34 (**Figures 1C, D**) (Xu et al., 2025) and conclude with continued discussion of AVP1’s translational potential in crop improvement through both genetic engineering and crop breeding strategies. Throughout, we emphasize AVP1’s role as a regulated transporter with functional flexibility.

### Energy demands and source–sink coordination under stress

Proper partitioning of photosynthates from source tissues such as leaves to roots, and reproductive organs is important for plant growth and yield (Lemoine et al., 2013). This source–sink relationship must be judiciously regulated to meet developmental demands and environmental obstacles. Understanding the molecular systems that coordinate energy use and carbohydrate flow across tissues will improve crop resilience to salinity, nutrient deprivation and drought (Munns and Millar, 2023). One approach involves AVP1, a type I H^+^-PPase, which acts as a central hub linking proton gradients, PPi metabolism, and long-distance transport (Pizzio et al., 2017).

Photosynthetic leaves and other source tissues, produce and export sucrose via the phloem. Sink tissues, such as roots, emerging leaves, flowers, and developing fruits, are usually importers of carbon. In plants, often the sites of carbon production are separated from the sites of carbon utilization, necessitating long-distance transport through the phloem. The efficiency of this movement impacts not only plant biomass but also the ability to tolerate stress (Praveen et al., 2023).

Phloem loading can occur via symplastic or apoplastic means, and in most crop species, the apoplastic pathway predominates (Rennie and Turgeon, 2009). In this apoplastic route, sucrose is loaded into sieve elements by H^+^/sucrose symporters like SUC2, a process that requires a large proton gradient (Stein and Granot, 2019). Any mechanism that enhances the gradient can positively impact source–sink communication.

The strength of sink tissues changes during growth and development. Strong sinks require robust source tissues, stimulating sucrose export. AVP1 responds to changing sink/source demands: energizing phloem transport and supporting anabolic metabolism in sink tissues (Shiratake et al., 1997). As discussed here, its dual function at both vacuolar and plasma membranes positions it to influence both ends of the transport equation.

Emerging evidence also suggests that source–sink coordination involves intricate feedback loops, including sugar signaling, hormonal crosstalk (notably involving auxin and cytokinin), and metabolic status indicators like Trehalose 6-Phosphate (T6P) (Sakr et al., 2018). T6P levels fluctuate with sucrose availability and influence growth-related gene expression. It is plausible that AVP1 activity, by modulating sucrose flux and PPi levels, indirectly affects T6P-regulated pathways (Gonzalez et al., 2010). Integrating AVP1 into this broader signaling landscape may help explain how its activity yields systemic changes in plant architecture and reproductive output.

Abscisic acid (ABA) has emerged as a key hormone modulating sink development and phloem unloading during stress (Mo et al., 2024). AVP1’s known role in drought resistance might involve ABA-responsive expression patterns although currently there is no clear mechanistic evidence (Gaxiola et al., 2001). Altering plant transporters has been shown to both directly and indirectly impact plant hormone levels (Magidin et al., 2003; Tabeta et al., 2021). Understanding how AVP1 influences or responds to hormone gradients may identify new regulatory entry points for engineering stress-resilient crops.

### Functions of H^+^-PPases at the vacuolar membrane

At the tonoplast, AVP1 hydrolyzes PPi to drive proton accumulation in the vacuole (**Figure 1A**). This acidification is essential for ion sequestration, detoxification, pH homeostasis, and storage of secondary compounds (Maeshima, 2000). The vacuolar H^+^-PPase also reduces cytosolic PPi, a byproduct of numerous biosynthetic reactions, thereby promoting anabolic fluxes such as gluconeogenesis and cellulose biosynthesis (Ferjani et al., 2011; Martinoia et al., 2007).

These functions are particularly important in meristematic and rapidly growing tissues where high biosynthetic activity generates abundant PPi and ATP supply may be limited (Shiratake et al., 1997). AVP1 activity contributes to sink strength by supporting growth under both normal and stress conditions. Transgenic studies have demonstrated that AVP1 overexpression improves root architecture, enhances biomass accumulation, and increases tolerance to salinity and phosphate starvation (Gaxiola et al., 2012; Heuer et al., 2017; Yang et al., 2007). These results establish AVP1 as a viable target for manipulating plant vigor and metabolic efficiency.

Further proteomic and transcriptomic studies should be initiated to map out the broader network of proteins and genes affected by H^+^-PPase activity. For example, given the phenotypic data it is easy to envision that AVP1-overexpressing plants display altered expression of ion transporters, antioxidant enzymes, and metabolic regulators (Pizzio et al., 2015). These detailed expression studies could further define how AVP1 serves as a master regulator of cellular homeostasis, influencing a cascade of downstream pathways.

## A second location: AVP1 at the plasma membrane

Evidence reveals that AVP1 is not confined to the vacuole (**Figure 1A**). It is also found at the plasma membrane, especially in phloem companion cells and possibly in roots (Jones et al., 2014; Pizzio et al., 2015). At the plasma membrane, AVP1 can function in reverse mode, synthesizing PPi from Pi using the proton gradient maintained by H^+^-ATPases. This reverse activity has been demonstrated through patch-clamp studies and yeast vesicle reconstitution (Scholz-Starke et al., 2019). In companion cells, reverse-mode AVP1 activity provides PPi to drive sucrose hydrolysis via Sucrose Synthase, supporting downstream ATP production and powering sucrose loading into sieve elements via SUC2. The generated ATP also fuels H^+^-ATPases, maintaining the steep proton gradient required for H^+^/sucrose symport. This feedback loop enhances phloem loading capacity and supports long-distance transport (Khadilkar et al., 2016; Pizzio et al., 2015; Regmi et al., 2016a).

In roots, AVP1 at the plasma membrane may support nutrient uptake and membrane potential stability under stress. Immunolocalization studies in wheat and rice show AVP1 expression in vascular tissues and developing sinks, indicating a conserved role in coordinating carbon flow and osmotic regulation (Regmi et al., 2020, 2016b).

The dynamic localization of AVP1 suggests a regulatory mechanism that targets the enzyme to different membranes depending on developmental stage, cell type, or environmental condition. Advanced imaging techniques and proteomic profiling of subcellular fractions will be key to understanding how this targeting is achieved.

### Genetic evidence for AVP1’s role in phloem loading

Transgenic Arabidopsis lines overexpressing AVP1, either constitutively or under companion cell-specific promoters, exhibit increased photosynthesis, rosette size, root and shoot biomass, and enhanced sucrose translocation. These traits point to AVP1’s importance in phloem function and metabolic integration. Crucially, companion cell-specific expression isolates AVP1’s effect within the phloem-loading context, demonstrating that systemic phenotypes are not mere artifacts of general metabolic upregulation (Khadilkar et al., 2016; Pizzio et al., 2015).

In wheat, AVP1 is localized to the plasma membrane of sieve element–companion cells. Transgenic wheat expressing Arabidopsis AVP1 show increased 14C-sucrose partitioning to roots, improved grain yield, and greater root biomass in greenhouse and field environments. These findings confirm AVP1’s central role in enhancing source–sink carbon transport in different crops (Regmi et al., 2020).

Additional studies in tomato and cotton are consistent with the findings that AVP1 modulates source/sink dynamics. For instance, AVP1-overexpressing tomato lines show rapid flowering and increased fruit set under drought stress (Yang et al., 2014). In cotton, overexpression lines display improved root systems and higher boll numbers (Pasapula et al., 2011). Together, these studies continue the broad research findings that AVP1 enhancement promotes not just vegetative vigor but reproductive success across different species.

Importantly, field trials of AVP1or its homologues-overexpressing lines under variable environmental conditions demonstrate that the observed phenotypes are stable and agronomically relevant (**Figure 1B**). For example, in semi-arid environments, transgenic corn and cotton plants expressing an AVP1 homologue maintained greater canopy biomass and reproductive output despite episodic water limitation (Li et al., 2008; Lv et al., 2009). These findings reinforce the case for AVP1 as a translatable target in diverse crop systems.

### Post-translational regulation of AVP1 by UBC34

AVP1 activity is subject to post-translational regulation, allowing plants to adjust their transport and metabolic capacity. One key regulator is UBC34, an E2 ubiquitin-conjugating enzyme that promotes AVP1 turnover at the plasma membrane (**Figure 1C**) (Xu et al., 2025). When UBC34 function is lost, as in ubc34 mutants, AVP1 accumulates at the membrane, leading to enhanced photosynthesis, increased sucrose levels in the phloem exudate, greater starch accumulation in leaves, and larger rosette diameter and biomass—traits that strongly resemble AVP1 overexpression lines (**Figure 1D**) (Xu et al., 2025).

These observations reinforce the concept that AVP1 is a limiting factor in determining source strength and phloem transport efficiency. The fact that SUC2 transcript levels also increase in *ubc34* mutants suggests a broader metabolic reprogramming in which enhanced AVP1 activity supports or even enables transcriptional upregulation of sucrose transport machinery (Xu et al., 2020). However, overexpression of SUC2 alone, without corresponding AVP1 support, fails to produce similar phenotypes and often results in growth defects (Dasgupta et al., 2014). This contrast emphasizes AVP1’s central role in buffering the energy costs of transport, facilitating ATP production via PPi-fueled glycolysis, and maintaining the proton gradients that energize H^+^/sucrose symporters like SUC2 (Lerchl et al., 1995).

Moreover, AVP1’s influence extends beyond the phloem. In *ubc34* mutants, elevated AVP1 levels at the plasma membrane may support broader metabolic stabilization under high flux conditions (Xu et al., 2025). This includes fueling biosynthetic reactions, maintaining membrane potential, and reducing cytosolic PPi buildup. These functions are critical in rapidly growing tissues, and during recovery where transport demand and metabolic activity are high.

The AVP1 phenotypes observed in ubc34 mutants suggest that plants tightly regulate AVP1 localization and stability to fine-tune carbohydrate loading and export (Xu et al., 2025). Rather than acting as a static enzyme, AVP1 serves as a modulator of source–sink interactions, adjusting phloem transport dynamics based on needs. Future efforts to manipulate AVP1 for crop improvement may benefit from targeting its regulatory circuits, such as modulating degradation under stress conditions, rather than relying solely on constitutive overexpression.

This UBC34 regulatory finding underscores that AVP1 is a key integrator of transport, energy homeostasis, and developmental plasticity. Its ability to influence both physiological traits and transcriptional outputs makes it a powerful lever for improving plant performance across environments (Gonzalez et al., 2010).

This work with UBC34 is transformative because it changes the way we approach future research regarding AVP1. Rather than speculating on localization or overexpression effects, this work established that AVP1 plasma membrane stability is modulated in response to metabolic demands. What’s enjoyable about this insight is that this work didn’t come from the AVP1 research community but from a group focused on SUC2 and sugar transport (Xu et al., 2020, 2025), another example of how the transport field often gets pushed forward by people thinking about metabolism. We’re now in new terrain where we can move past the point of asking whether AVP1 shows up at the plasma membrane—and now pose more interesting questions: how AVP1 gets there, when it’s needed, and what’s controlling it once it’s there. It’s reasonable to speculate that beyond targeted degradation, things like trafficking pathways, post-translational modifications, and interactions with other proteins are also shaping when and how AVP1 operates at the plasma membrane.

## Agricultural relevance: natural and engineered modulation of AVP1

AVP1 overexpression across a range of plant species including Arabidopsis, tomato, rice, maize, and cotton has demonstrated improvements in root and shoot growth, stress resilience, and reproductive output under a variety of growth conditions (Gaxiola et al., 2012). These phenotypes are attributed to improved phloem loading, greater sink strength, efficient nutrient acquisition, and efficient utilization of pyrophosphate (PPi). Under drought, salinity, and phosphate-starvation stress, AVP1-overexpressing plants show increased turgor, larger root systems, and higher harvest indices (Gaxiola et al., 2016a, b).

Notably, these beneficial traits extend beyond the lab. Field trials of AVP1-expressing lines have confirmed their agronomic relevance, showing stable yield advantages in water-limited and low-input soils (Paez-Valencia et al., 2013). For example, cotton plants overexpressing AVP1 maintain boll production and deeper rooting profiles under dryland farming conditions (Pasapula et al., 2011). These studies indicate that AVP1-based strategies can provide practical benefit, offering a combination of metabolic efficiency and physiological adaptability.

Natural genetic variation also plays a role in modulating AVP1 homologue activity and expression. In maize, a 366-bp insertion in the ZmVPP1 promoter introduces MYB transcription factor binding sites, upregulating expression and conferring increased drought tolerance in seedlings (Wang et al., 2016). This example shows that cis-regulatory variation can be harnessed without the need for transgenic technologies. In wheat, high-biomass cultivars such as Buck Atlantico and Scout exhibit elevated expression of the TaVP4B H^+^-PPase homolog, suggesting that natural selection or traditional breeding may have favored alleles enhancing H^+^-PPase activity (Menadue et al., 2021). These findings broaden the scope of AVP1 to include a breeding target beyond genetic engineering.

As mentioned previously, promoter engineering has been utilized to modulate AVP1 expression. AVP1 activity in the phloem impacts sucrose loading and long-distance transport. Similarly, combining AVP1 overexpression with transcriptional regulators can improve both energy utilization and stress perception. Modulating regulators like UBC34 through genome editing can also offer control by adjusting AVP1 protein stability.

Genome-wide association studies (GWAS) can identify changes in AVP1 expression and localization that improve yield. These beneficial variants can be breed into crops. Combining AVP1-enhancing alleles with other traits may also produce synergistic phenotypes.

### Broader implications for crop improvement

AVP1 or homologues can functions as tunable metabolic switches. Because it can function at two different membranes and move in either direction, it offers several ways to engineer plants. By enhancing phloem loading, maintaining PPi homeostasis, and supporting root architecture, AVP1 can improve both source and sink capacity through precise control of the regulatory switches.

Future crop improvement strategies can focus on condition-specific AVP1 stabilization, for example by modulating UBC34. Such targeted interventions would avoid the drawbacks of constitutive overexpression and maximize trait benefits where and when they are needed.

Because type I H^+^-PPases are conserved, these approaches should be applicable to many crops. However, it will be interesting to continue to compare and contrast results from monocots and dicots. Expanding our understanding of how AVP1 regulation is coordinated with other transporters will further refine its deployment. For example, upregulation of AVP1 and nutrient transporters might synergistically improve nitrogen and phosphor use efficiency in staple crops (Hirschi, 2008). Additionally, targeting AVP1 expression and stability during specific developmental stages will allow for temporal fine-tuning of carbon partitioning and yield improvement.

## Conclusion: AVP1 in energy and carbon partitioning

Decades ago, we opined about AVP1’s role in plant growth and development from a tonoplast centric viewpoint (Gaxiola et al., 2002). Today, these same phenotypes are explained more convincingly by dual localization, directional transport flexibility, and sensitivity to post-translational regulation. In the phloem AVP1 maintains proton gradients and supplies PPi for biosynthesis and glycolysis. In sink tissues, its vacuolar H^+^-pumping activity promotes biosynthetic processes and improves sink strength. These functions facilitate sugar transport and support plant growth and resilience. Future efforts to manipulate AVP1 stability and localization, rather than the current trend of altering expression alone, offer more precise strategies to optimize plant performance under diverse conditions.
